# Expanding National‐Scale Wildlife Disease Surveillance Systems With Research Networks

**DOI:** 10.1002/ece3.71492

**Published:** 2025-06-11

**Authors:** Kim M. Pepin, Matthew A. Combs, Guillaume Bastille‐Rousseau, Meggan E. Craft, Paul Cross, Maria A. Diuk‐Wasser, Roderick B. Gagne, Travis Gallo, Tyler Garwood, Jonathon D. Heale, Joshua Hewitt, Jennifer Høy‐Petersen, Jennifer Malmberg, Jennifer Mullinax, Laura Plimpton, Lauren Smith, Meredith C. VanAcker, Jeffrey C. Chandler, W. David Walter, Grete Wilson‐Henjum, George Wittemyer, Kezia Manlove

**Affiliations:** ^1^ National Wildlife Research Center, Wildlife Services, Animal and Plant Health Inspection Service United States Department of Agriculture Fort Collins Colorado USA; ^2^ Cooperative Wildlife Research Laboratory Southern Illinois University Carbondale Illinois USA; ^3^ Department of Ecology, Evolution, and Behavior University of Minnesota St. Paul Minnesota USA; ^4^ U.S. Geological Survey Northern Rocky Mountain Science Center Bozeman Montana USA; ^5^ Department of Ecology, Evolution, and Environmental Biology Columbia University New York City New York USA; ^6^ Department of Pathobiology, Wildlife Futures Program University of Pennsylvania School of Veterinary Medicine Kennett Pennsylvania USA; ^7^ Department of Environmental Science and Technology University of Maryland College Park Maryland USA; ^8^ Wildlife Services, Animal and Plant Health Inspection Services United States Department of Agriculture Fort Collins Colorado USA; ^9^ Department of Wildland Resources and Ecology Center Utah State University Logan Utah USA; ^10^ Global Health Program Smithsonian National Zoo and Conservation Biology Institute Washington DC USA; ^11^ U.S. Geological Survey, Pennsylvania Cooperative Fish and Wildlife Research Unit The Pennsylvania State University University Park Pennsylvania USA; ^12^ Department of Fish, Wildlife and Conservation Biology Colorado State University Fort Collins Colorado USA

**Keywords:** chronic wasting disease, disease emergence, mule deer, opportunistic surveillance, SARS‐CoV‐2, severe acute respiratory syndrome coronavirus 2, surveillance, targeted surveillance, white‐tailed deer, wildlife disease

## Abstract

Efficient learning about disease dynamics in free‐ranging wildlife systems can benefit from active surveillance that is standardized across different ecological contexts. For example, active surveillance that targets specific individuals and populations with standardized sampling across ecological contexts (landscape‐scale targeted surveillance) is important for developing a mechanistic understanding of disease emergence, which is the foundation for improving risk assessment of zoonotic or wildlife‐livestock disease outbreaks and predicting hotspots of disease emergence. However, landscape‐scale targeted surveillance systems are rare and challenging to implement. Increasing experience and infrastructure for landscape‐scale targeted surveillance will improve readiness for rapid deployment of this type of surveillance in response to new disease emergence events. Here, we describe our experience developing and rapidly deploying a landscape‐scale targeted surveillance system for severe acute respiratory syndrome coronavirus 2 (SARS‐CoV‐2) in two free‐ranging deer species across their ranges in the United States. Our surveillance system was designed to collect data across individual, population, and landscape scales for future analyses aimed at understanding mechanisms and risk factors of SARS‐CoV‐2 transmission, evolution, and persistence. Our approach leveraged partnerships between state and federal public service sectors and academic researchers in a landscape‐scale targeted surveillance research network. Methods describe our approach to developing the surveillance network and sampling design. Results report challenges with implementing our intended sampling design, specifically how the design was adapted as different challenges arose and summarize the sampling design that has been implemented thus far. In the discussion, we describe strategies that were important for the successful deployment of landscape‐scale targeted surveillance, development and operation of the research network, construction of similar networks in the future, and analytical approaches for the data based on the sampling design.

## Graphical abstract

Pathogen transmission in wildlife populations depends on ecological factors that feed back across the scales of individuals, populations, and landscapes. We developed and deployed a disease surveillance design for aligning data collection across these multiple biological scales (landscape‐scale targeted surveillance) to enable future analyses to identify ecological drivers of disease transmission and predict hotspots of disease emergence in wildlife. We describe our experience with the deployment of landscape‐scale targeted surveillance of severe acute respiratory syndrome coronavirus 2 (SARS‐CoV‐2) in two free‐ranging deer species across their ranges in the United States. We identify strategies that are important for successful deployment of landscape‐scale targeted surveillance in wildlife using a research‐based surveillance network.

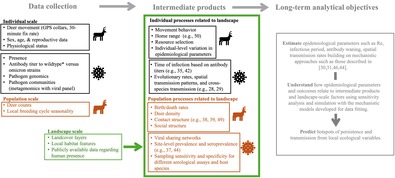

## 1 Introduction

Protecting human, livestock, and wildlife populations from pathogen transmission from wildlife reservoirs depends on effective surveillance systems at regional, national, and international scales (Holmes et al. 2019; Pedersen et al. 2012; Sokolow et al. 2019; Watsa 2020; World Organisation for Animal Health and IUCN 2023). Active surveillance generally provides the best information because it is designed intentionally with specific objectives in mind. Active surveillance systems often target specific hosts or pathogens and may operate at broad geographic scales across ecological contexts (World Organisation for Animal Health and IUCN 2023; Bevins et al. 2022; Gervasi et al. 2019; Schatz et al. 2014), which is important for understanding and predicting disease transmission in wildlife. The spatial design of active surveillance systems influences the type of intelligence that can be obtained (Table 1). A primary distinction in active surveillance systems is whether the spatial and temporal design of sampling is tied to preexisting human‐wildlife activities (such as management or hunting; opportunistic sampling (World Organisation for Animal Health and IUCN 2023; Bevins et al. 2022; Gervasi et al. 2019; Schatz et al. 2014)) or whether the design is developed to target specific individuals or population‐level disease transmission processes (targeted sampling).

**TABLE 1:** Types of sampling designs used in active wildlife disease surveillance systems and their suitability for providing different wildlife disease insights across biological scales of inference (Individual—I, Population—P, Landscape—L). The ability of each sampling design to provide robust inference at each scale is indicated with either an open circle (not suitable, poor inference), half‐circle (suitable but not ideal), or closed circle (highly suitable, robust inference). Clarifying details are provided for certain cases where needed.

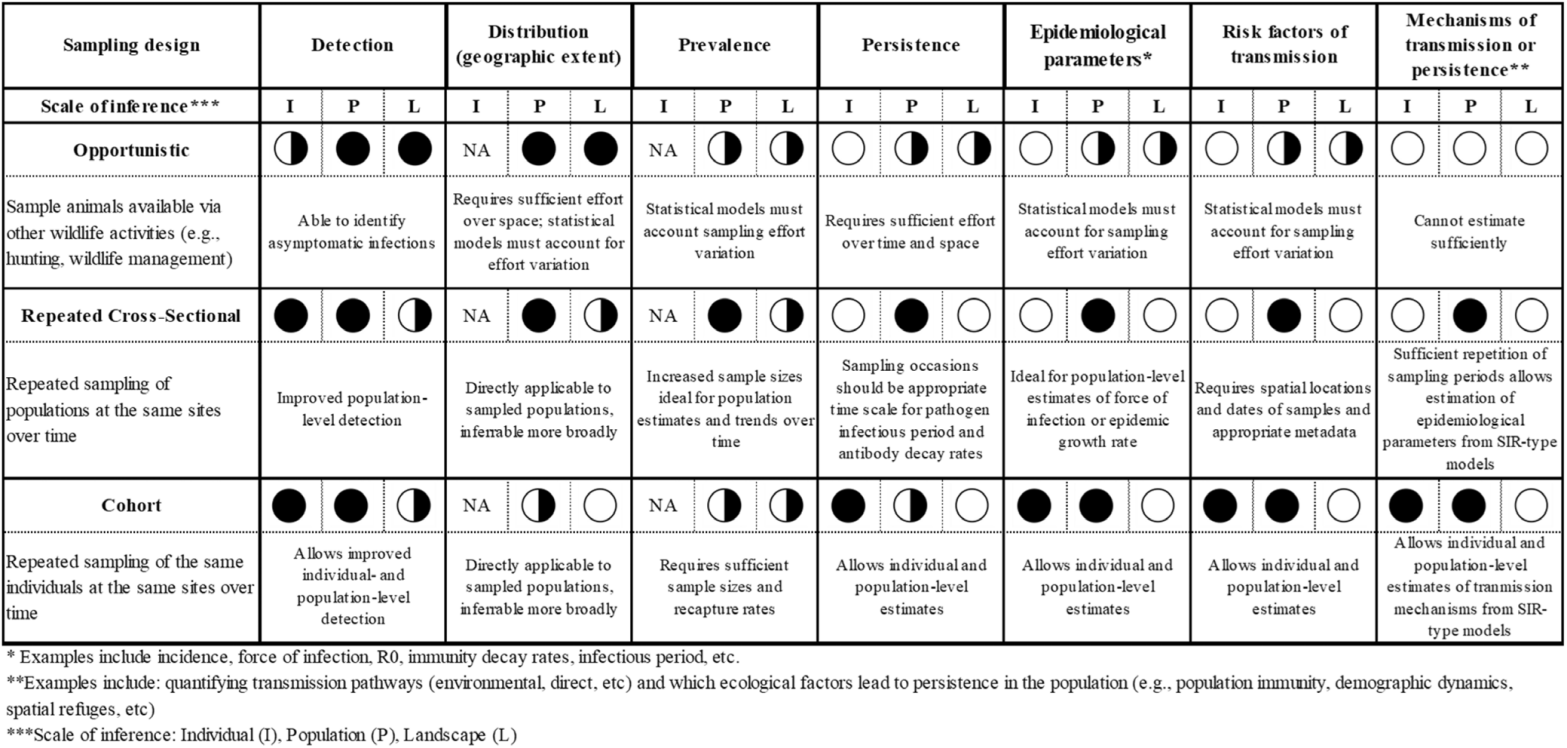

Opportunistic sampling is used more commonly across ecological contexts than targeted sampling because opportunistic sampling can leverage preexisting infrastructure to facilitate land access, animal capture, and sampling. However, opportunistic sampling is most beneficial for characterizing the spatial distribution of disease occurrence (Table 1). Variation in metadata quality, spatial coverage and depth, and capacity to target specific population processes can make it difficult to infer epidemiological parameters or ecological risk factors from opportunistic sampling. In contrast, a targeted sampling design can address these gaps, especially when it is structured to understand population processes at a specific study site and replicates the same sampling design across study sites. To that end, targeted sampling could involve a sampling design that allows coordination of data collection across individual, population, and landscape scales (Walsh and Miller 2010). Two common targeted sampling designs in epidemiology are repeated cross‐sectional sampling (repeated sampling of different individuals in the same population) (Hazel et al. 2000; Vosloo et al. 2009; Telfer et al. 2008) and cohort sampling (repeated sampling of the same individuals in the same population) (Alasaad et al. 2013; Duffus et al. 2022; Delahay et al. 2000) (Table 1). In both sampling designs, host behavior, population demographics, and other appropriate metadata can be combined with disease diagnostic results to understand factors that increase the risk of disease transmission, establishment, and persistence in wildlife populations.

Individual‐level cohort sampling has long been recognized as a gold standard for understanding and predicting ecological dynamics (Clutton‐Brock and Sheldon 2010). The particular strength of a cohort study is its information on how individual status changes through time (i.e., individual transitions from susceptible to infectious to recovered—the determinants of outbreak trajectories). Similar estimates can be acquired through experimental infections, but those estimates may be biased by artificial conditions due to containment and sourcing of individuals (e.g., captive reared individuals are usually all exposed to similar captive conditions). In the context of disease ecology, following infection trajectories through time within individuals in natural populations provides more accurate estimates across individuals in nature (e.g., (Plowright et al. 2017; Baker et al. 2014)) for predicting outbreak trajectories. The fundamental weaknesses of cohort sampling are its cost (it is generally more expensive to recapture and resample specific individuals than it is to gather a new random sample without considering testing history) and its ability to cover the full array of heterogeneity within the system (due to limitations on sample sizes). Cross‐sectional sampling at the population level is cheaper and easier to implement than cohort sampling but is limited to providing information about host disease states within individuals at one point in time. This makes estimates of state‐transition rates from cross‐sectional sampling more uncertain than those from cohort sampling (Lambert et al. 2022).

A powerful design for understanding emergence of a new pathogen in a new host species is to conduct parallel cohort sampling across multiple populations in different ecological contexts (hereafter termed ‘landscape‐scale targeted surveillance’), and to combine those data with cross‐sectional sampling conducted across a broader array of individuals and populations (Réveillaud et al. 2018). This leverages strengths from both cohort and cross‐sectional sampling and can illuminate how individual‐level and population‐level factors combine to determine transmission dynamics in different environmental conditions. Landscape‐scale targeted surveillance has been beneficial for uncovering the mechanistic drivers and emergent patterns of shifting plant phenology (Tang et al. 2016), and addressing critical drivers of biological invasions (Gill et al. 2021). However, while the value of these data are recognized in wildlife disease systems, the logistical challenges of setting up a landscape‐scale targeted surveillance system that targets particular individuals and populations over time in wildlife, means that they are rarely operationalized in practice.

Because landscape‐scale targeted surveillance is labor‐intensive and interdisciplinary, effective development and deployment may benefit from leveraging the strengths of diverse partners across public service and public‐private research institutions. To that end, we built and deployed a research network to conduct landscape‐scale targeted surveillance for SARS‐CoV‐2 in two cervid species, mule deer (MuD, *Odocoileus hemionus* ) and white‐tailed deer (WTD, *Odocoileus virgianus*), throughout the United States (www.targetedsurveillance.com). The surveillance system was developed to provide data that could address the following long‐term objectives: (1) understand epidemiological risk factors for the emergence of SARS‐CoV‐2 and other pathogens across the range of wild deer, (2) predict landscape‐level hotspots of disease emergence across the range of wild deer, and (3) advance methods for predicting spatial disease dynamics in wildlife populations by scaling up wild deer movement data in different geographical contexts to population‐level disease dynamics.

**FIGURE 1:**
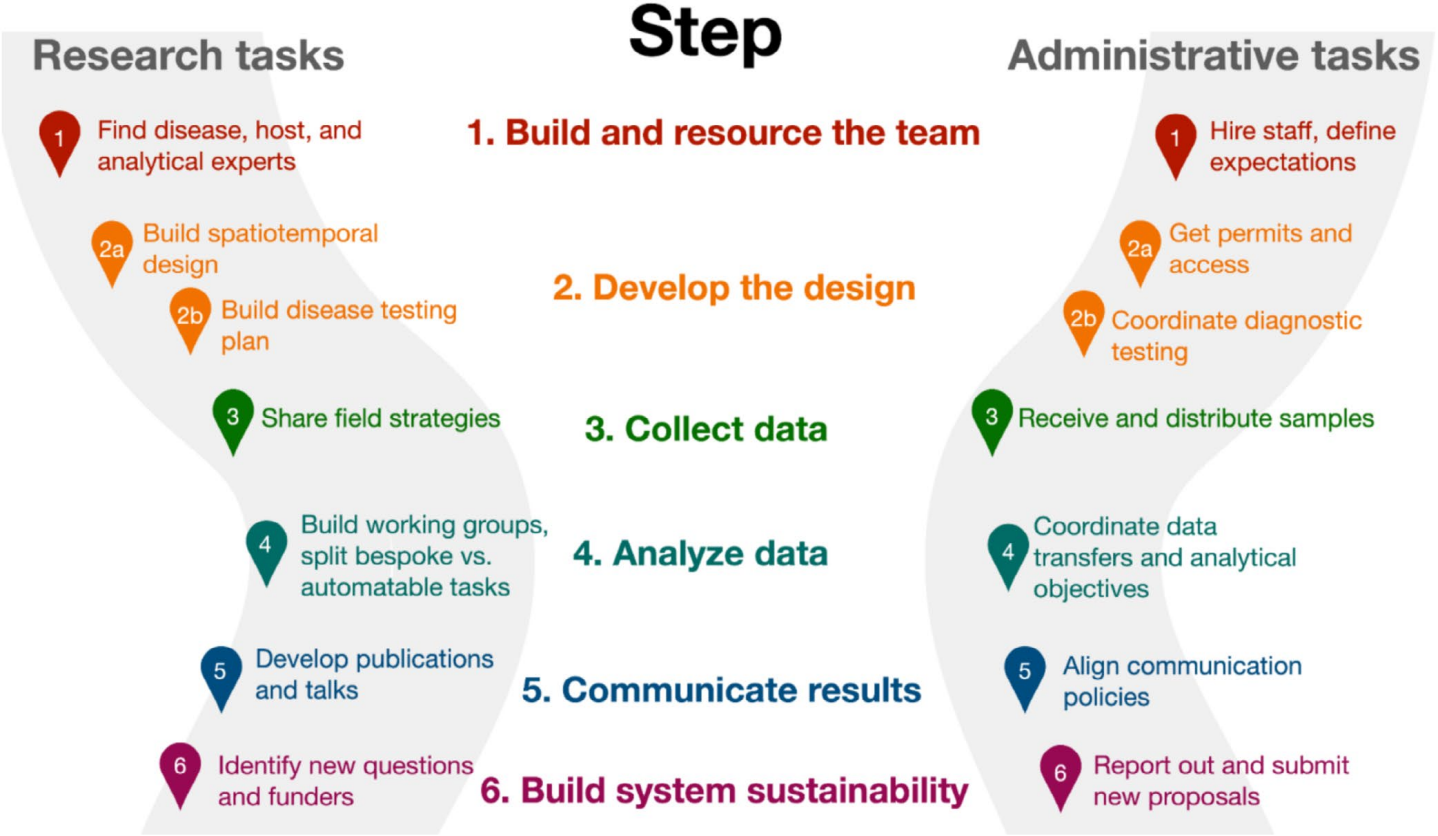
Steps for building a landscape‐scale targeted surveillance research network. Colors link research and administrative tasks to specific steps in network construction. Wide gray lines indicate sequential workflows in the Research and Administrative domains.

Here, we provide an after‐action review (i.e., a collective self‐examination of the surveillance deployment (Morrison and Meliza 1999; Cronin and Andrews 2009)) of a necessary antecedent to those objectives, namely, establishing the network and designing and implementing the surveillance scheme itself (Figure 1). In the Methods, we describe the network's construction and the planned study design; in the Results, we describe deviations from and alignments with the planned design, techniques that were actually implemented, and a brief summary of data collected thus far. The Discussion identifies elements that led to successful deployment and offers insight about how to overcome implementation challenges.

## 2 Methods

### 2.1 Study System

During the early stages of detection of severe acute respiratory syndrome coronavirus 2 (SARS‐CoV‐2) in wild WTD in the United States (Chandler et al. 2021), a national‐scale surveillance system using an opportunistic sampling design was rapidly deployed starting in fall 2021 through voluntary participation from state agencies and federal partners using established networks of United States Department of Agriculture's (USDA) Animal and Plant Health Inspection Service (APHIS), Wildlife Services (WS), and National Wildlife Disease Program (NWDP) (Bevins et al. 2023). It soon became clear that both WTD (Martins et al. 2022) and MuD (Porter et al. 2024) could maintain active infections of SARS‐CoV‐2 and transmit the virus among conspecifics, and that cross‐species transmission among humans and deer can and does occur (Pickering et al. 2022; Feng et al. 2023). However, the opportunistic sampling design left several key knowledge gaps, including a mechanistic understanding of how ecological factors determine: (1) within‐population transmission, (2) spatial dynamics of SARS‐CoV‐2 invasion, and (3) the duration that SARS‐CoV‐2 variants can persist within cervid populations. Addressing these gaps requires a sampling design that tracks individuals and populations according to a similar sampling design applied in different ecological contexts (landscape‐scale targeted surveillance).

### 2.2 Building the Targeted Surveillance Research Network

In April 2019, a subgroup of seven wildlife disease and movement ecologists began collaborating on methods development at the movement‐disease interface (Manlove et al. 2022; Wilber et al. 2022). Following the detection of SARS‐CoV‐2 in white‐tailed deer, we hypothesized that variation in wild deer spatial ecology would contribute to SARS‐CoV‐2 transmission and reservoir potential in wild deer. Beginning in April 2022, we began developing and proposing implementation plans for a surveillance system that could test that hypothesis while also delivering appropriate data for spatial risk assessment and epidemiological modeling of SARS‐CoV‐2 in wild deer. The sampling design was motivated by our previous modeling work (Manlove et al. 2022; Wilber et al. 2022; Pepin et al. 2017) and that of other mechanistic epidemiological modeling approaches that integrate wildlife spatial ecology and disease transmission (Dougherty et al. 2018; Albery et al. 2021; Merkle et al. 2018).

The first step, completed in late spring 2022, was to propose a site‐level, itemized budget to understand how many states/institutions could be included as principal investigators (PIs) (Figure 1). The initial rationale was for the budget to be enough for each state to implement the study design in at least one site in the first year, realizing that PIs with preexisting projects involving global positioning system (GPS)‐collared deer may be able to establish more sites. The site‐level budget was determined through structured conversations about the different types of capture techniques (e.g., helicopter captures, ground darting, aerial drop nets, Clover traps) available at different potential sites, different GPS radiocollar costs, capture supply costs, and personnel time needed. We then took an average of the differences to create a prototypical standardized site‐level budget. The number of potential PIs was then determined by dividing the total potential budget by the prototypical site‐level budget. Funding was allocated equally to all sites, but one institution received additional funding for support staff that coordinate network administration, communication, diagnostics, supply ordering, and sample shipping and receiving across the research network. These positions are colocated with the public sector partners and diagnostic lab.

Initial funds were approved for distribution through cooperative agreements to research institutions beginning October 1st, 2022, at the 10% indirect cost rate required by the funder. This coincided with hunting seasons in some sites. Thus, repeated cross‐sectional sampling was implemented within a month of receiving funding approval in sites with ongoing hunting. It took approximately 2 months for all seven initial institutions to finalize funding agreements for spending due to differences in institution staffing, communication, and experience with the type of agreement. Some GPS collaring manufacturers require up to 4 months from the time of receiving a large collar order to shipment to the customer, and these supply chains were backed up during the COVID‐19 pandemic. Thus, sites that already had some collars on hand from ongoing work were able to get started earlier. Start times for cohort sampling ranged from mid‐November 2022 until February 2023 (up to 4.5 months following approval of funding).

Using our scientific network and budget planning information, the subgroup of PIs identified two additional collaborators during the summer of 2022 who had experience working on wild deer across diverse ecological contexts ranging from rural to suburban or urban to form the complete research network. Desired characteristics for research network members included expertise in ungulate ecology, ungulate capture and handling, animal movement ecology, disease ecology and epidemiology, disease surveillance design and statistics, viral diagnostics, and viral genomics, experience with administering and managing large research programs, and the ability to begin capture and sampling immediately. Another key characteristic was the desire and behavioral disposition to effectively conduct team science—specifically, willingness to share data among the team members and allow a variety of members to serve as leads or senior authors on the broader multicontributor dataset. Rules for data sharing were established through structured conversations, and a multi‐institution data sharing agreement was developed during October–November 2022 and signed by all institutions by March 5th 2024. The data sharing agreement specified that GPS data are generally owned by the institutions that collected them but may be shared among institutions or publicly upon request, following guidelines in the data sharing agreement. Because state wildlife agencies have jurisdiction with deer management, release of fine‐scale spatial information about individual deer locations and disease status is sensitive and must be requested from both the institution that collected the data and the state wildlife agency. Once the initial research network was established, two other research teams joined using independent funding sources and applied the surveillance design guidance and data sharing protocols that the network developed (described below). Those institutions became aware of the work through scientific networks and joined because they recognized value in leveraging their data for landscape‐scale inference. This brought the total network size to eight academic institutions and two federal agencies, distributed across nine states. Sampling commenced at all sites over the winter of 2022–2023.

### 2.3 Coordination and Internal Processes

Regular, structured communication has been vital during all stages—planning, deployment, and data management and analysis. We began with weekly virtual meetings among institution PIs that decreased in frequency to bi‐weekly or monthly once the network was established, along with a network‐wide (all personnel) in‐person annual meeting. We designated a coordinator to lead meetings, plan agendas, and manage funding agreements.

During the project's first 6 months, meetings were structured around communication strategies including the development of a website (targetedsuveillance.com), coordinating oral presentations, media communications, and a central repository for information sharing (e.g., surveillance data, meeting agendas, sampling protocols, and sample storage and shipping information). We designated a staff member to order and ship supplies to all states, receive samples, coordinate with the SARS‐CoV‐2 diagnostic laboratories, collate and manage diagnostic data, plan additional supply orders, and archive samples. This centralized approach facilitated the standardization of sample collection, storage, and data management for the diagnostic data. We also established a subgroup that developed standardized GPS data cleaning protocols (i.e., verifying collar start and end dates, making projection information consistent, applying a standardized protocol for identifying and removing GPS fix errors, etc.) and hired personnel to implement the protocols and work with individual states to address site‐level nuances.

A second major topic of initial meetings was the development of the sampling design and how it could be standardized given realities at different sites. Through structured discussions about ideal design elements (described below), these meetings delivered potential contingency actions that sites could implement to keep the design as standardized as possible. These meetings also provided opportunities for network members to share solutions to implementation challenges as they arose, increasing the efficiency of implementation for other sites. Once all network members had well‐established sites with effective capture and sampling occurring, the meetings shifted toward planning analytical workflows and hiring appropriate staff.

**FIGURE 2:**
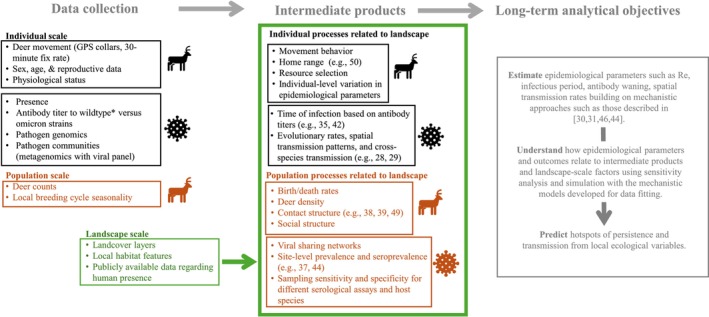
Schematic of the planned analytical products. Colors of the text distinguish data types and products at different ecological scales (individual, population, landscape). Icons represent whether the data describe host (deer) versus pathogen (virus) processes. The green box around data products indicates that these processes are being estimated across different ecological contexts.

Analyses (Figure 2) will primarily be conducted by a team of PhD students and post‐doctoral researchers that coordinate as a team, in subgroups, and with PIs. To reduce potential redundancy and standardize analyses, we implemented the following workflows: (1) anyone wishing to lead a data analytic component that aligns with our surveillance system objectives must propose their specific aims and data requirements in a meeting with PIs and receive approval by email from all PIs, (2) analysts attend appropriate subgroup meetings with other personnel that need similar data or intermediate data products to share tasks and standardize the production of data products, and (3) analysts present progress on their work at regular PI meetings and annual meetings.

### 2.4 Overview of the Targeted Surveillance Approach

Our sampling design operates across individual, population, and landscape scales. At the individual level, our objective was to collect data to understand disease progression (e.g., infection rate, infectious period, propensity for chronic carriage, antibody kinetics) within individual hosts, as well as host movement. At the population level, our objective was to collect data to understand how host density, social structure, and contact processes on real landscapes drive local patterns of pathogen spread. At the landscape scale, our objective was to select sites that varied in local habitat features, human density, and landcover so that we could understand how the risk of SARS‐CoV‐2 establishment and persistence in deer populations relates to ecological context. Thus, we aimed to select study populations across an urban–rural gradient and across multiple ecosystems where SARS‐CoV‐2 was predicted to occur in WTD (Hewitt, Wilson‐Henjum, Collins, Linder, et al. 2024) or suspected to occur in MuD (Table 2). While we did not set a priori strata that had to be fulfilled by our sites, we considered how well our sites covered the rural–urban gradient qualitatively throughout the research network formation phase. In retrospect, our sites varied substantially in human population density, from very dense Staten Island and Maryland/District of Columbia sites to agriculturally centered, low‐human‐density sites in Colorado, Tennessee, and Illinois, to several very low‐density and largely natural lands sites in Utah.

**TABLE 2:** Description of differences among sites in sample collection and local ecology.

State institution	Host species	Site	FY	Site area (km^2^)	Capture period	Preexisting work	Land ownership	Urbanity	Ecotype (level II: level III)^b^	Site habitat	Capture method	No. individuals with GPS collars	Total collar days	Samples from harvested animals	Samples from GPS collared animals	Recaptures
Colorado	MuD/WTD	1 (Platte)	2023	100	February 2023–May 2023	N	Private	Suburban	South central semiarid prairies: high plains	Suburban development, industrial livestock (feedlots), dairy production, & natural gas developments	Clover trapping, ground darting, drop netting	13	2515	0	20	1
Colorado state			2024		January 2024–May 2024							10	1350	0	24	4
University		2 (Poudre)	2023	80	April 2023–May 2023; October–December 2023	N				Suburban development, livestock & dairy production		41	1511	0	39	5
			2024		January 2024–May 2024							51	2106	0	80	11
Illinois	WTD	1 (Shelbyville)	2023	261.9	January 2023–March 2023	Y	Private/Public (State)	Rural	Central plains: central corn belt plains	Predominently soy & corn farms	Clover trapping, ground darting, drop netting	24	4109	0	29	0
Southern			2024		December 2023–March 2024							23	3861	1	26	0
Illinois		2 (Touch of Nature)	2023	16.2	January 2023–March 2023	Y			Southeastern plains: interior river valleys and hills	Predominently forrested, exurban & agricultural development		37	5490	1	41	1
University			2024		January 2024–March 2024							36	5749	7	39	1
Maryland & Washington, D.C.	WTD	1 (DC)^a^	2024	50	January 2024–March 2024	N	Private/Public (Federal)	Urban	Southeastern plains: northern piedmont/southeastern plains	Highly urban, small parks & green spaces, some remnant natural forest cover	Drop netting	6	449	44	7	0
University of Maryland		2 (MD)^a^	2024		January 2024–March 2024		Public (Municipality)					13	1586	50	20	0
Minnesota	WTD	1	2023	42.1	January 2023–March 2023	N	Public (Municipality)	Suburban	Mixed wood plains: north central hardwood forests	Park with wetlands, forest, & prairie land cover, surrounded by suburban, exurban, & cropland development	Helicopter net gunning	42	13,069	68	74	33
University of			2024		January 2024							6	4422	0	6	0
Minnesota		2	2023	33.5	NA							NA	NA	0	0	0
			2024		January 2024							43	4436	56	56	0
		3	2023	41.9	NA		Private/Public (Tribal)					NA	NA	0	0	0
			2024		January 2024							26	2341	0	25	0
Nebraska NWRC & CSU	MuD	1	2024	9100	February 2024‐ongoing	N	Private/Public (State/Federal)	Rural	South central semiarid prairies: high plains	Native short‐grass prairie, interspersed ponderosa or juniper forest & cropland	Helicopter net gunning	52	3933	0	60	0
New York	WTD	1 (Aggregated)	2023	43	March 2023; August 2023	Y	Public (State and City)	Urban	Mississippi alluvial and southeast coastal plains: Atlantic coastal pine barrens	City parks of deciduous forest, grassland, & wetland, surrounded by highly urban	Ground darting	48	13,049	0	83	34
Columbia University			2024		January 2024							41	4754	0	28	28
Pennsylvania	WTD	1 (Bedford)	2023	487	March–April 2023	Y	Private/public (State)	Rural	Appalachian forests: ridge and valley	Agricultural lands, interspersed deciduous forest	Clover trapping, rocket and drop netting	35	5358	0	38	3
Pennsylvania			2024	980	January–March 2024							49	4095	0	49	7
State university		2 (Fulton)	2024	180	March‐24	N					Clover trapping, rocket and drop netting	12	500	0	12	0
		3 (Treasure Lake)	2024	36	January–March 2024, June–August 2024	N	Private	Suburban	Appalachian forests: central Appalachians	Gated suburban community with wetlands, forest, & outdoor recreation land use	Ground darting	40	2716	0	40	2
Tennessee	WTD	1 (Ames)	2023	183	January–April 2023	Y	Private	Rural	Southeastern plains: southeastern plains	Rural, forrested area, surrounded by crop agriculture research center	Clover trapping, ground darting, Helicopter net gunning^c^	19	6023	39	29	3
University of			2024		January 2024–April 2024							39		41	37	0
Tennessee		2 (Lone Oaks)	2023			N						NA	NA	0	0	0
			2024	21	February–April 2024							11	305	0	12	0
Utah	MuD	1 (La Sals)	2023	2267	December 2022–March 2023	Y	Public (Federal)	Rural	Cold deserts: Colorado plateaus	Rural wildlands, high outdoor recreation, some free ranging cattle, sagebrush or pinyon‐juniper forest.	Helicopter net gunning	62	31,869	0	118	25
Utah state			2024		December 2023, March 2024							66		0	123	43
University		2 (San Juan)	2023	4938	December 2022–March 2023	N						63	34,073	0	103	20
			2024		December 2023, March 2024							75		0	111	31
		3 (Nebo)	2023	2529	December 2022–March 2023	Y	Private/Public (Federal)	WUI	Western Cordillera: Wasatch and Uinta Mountains	Wildland‐urban interface, subalpine fir/Engelmann spruce forest ecosystem		72	32,284	0	101	15
			2024		December 2023, March 2024							80		0	135	44
		4 (Oquirrhs)	2023		NA	Y	Public (Federal)					NA	NA	0	0	0
			2024		December 2023							43		0	43	0
		5 (Pine Valley)	2023	3323	NA	Y			Cold deserts: central basin and range	Wildland‐urban interface, sagebrush or pinyon‐juniper forest.		NA	NA	0	0	0
			2024		December 2023, March 2024							60	8419	0	90	17
		6 (Range Creek)	2024	1228	December 2023, March 2024	Y	Private	Rural	Western Cordillera: Wasatch and Uinta Mountains	Rural, sagebrush or pinyon‐juniper forest		85	13,382	0	104	19

Abbreviation: FY, fiscal year.

^a^ These sites were in urban areas where patch size was too small to have contiguous sites. They were comprised of many small patches where animals may not contact one another.

^b^ Commission for environmental cooperation site.

^c^ This was added to other methods at the site during the second year of capture (2024) only.

### 2.5 Features of the Cohort Sampling Design

At each site, our goal was to capture at least 40 deer within a contiguous area (i.e., overlapping home ranges, as in (Yang et al. 2021)) to understand contact structure among individuals within an interacting population. We chose 40 deer based on our research network's experience with sample sizes that are adequate for estimating within‐population contact and disease transmission processes (Wilber et al. 2022; Yang et al. 2021, 2023), while accounting for the expense of GPS collars. Our goal was to track individuals from different social groups. In practice, this meant that we often tracked 2 or 3 individuals from the same group to minimize redundant data from animals moving together (as in (Yang et al. 2021)) while still allowing for mortalities or device loss/malfunction. We planned for GPS collar fix rates of 30‐min intervals from the first capture until the animal left the study. The 30‐min fix rate was selected based on our past experience extracting contact events from GPS data at coarser temporal scales in deer (Yang et al. 2023). Battery life was chosen to last 1–2 years to collect data across seasons, and we budgeted for annual collar replacement due to battery failure as needed. We planned to sample the same animals 1–3 times per year over the course of 2 years (a total of ~4–6 captures per animal over a two‐year period) and gather appropriate metadata (standardized across sites) and diagnostic samples at each capture event. We did not specify particular GPS collar manufacturers or models and allowed sites to choose their own vendors so that they could leverage preexisting contractual relationships. We did not plan density estimation methods before deployment began because: (1) most sites had preestablished methodology used by their state Department of Natural Resources (DNR), and (2) our budget was limited in some sites for deploying a standardized high‐resolution density estimation method. Each research team established separate but similar Institutional Animal Care and Use Committee protocols through their institution following group discussion of the research design (Colorado State University: #3872 and #5002 for Northern Colorado and Nebraska sites, respectively; Southern Illinois University: #21–028; University of Maryland: #FR‐AUG‐23‐37; University of Minnesota: #2209‐40440A; Columbia University: #AC‐AABT8667; Pennsylvania State University: #202202225; University of Tennessee Knoxville: #2850–1021; Utah State University: #13045).

### 2.6 Features of the Disease Testing Plan

The intent of longitudinal sampling at the individual level was to allow us to identify intervals in which specific individuals convert between disease states (e.g., susceptible, infected, recovered). In some sites we also sampled additional individuals that were removed during hunting or management to increase sample sizes describing disease status of the population (repeated cross‐sectional sampling). Because potential effects of age and sex on within‐host processes are poorly understood, we aimed to sample the sexes and age classes (yearling versus adult) in equal ratios. Sites were free to capture deer using any feasible technique given the local ecology, preexisting equipment and infrastructure, and alignment with state DNR practices. Consequently, sites chose from a variety of capture methods including helicopter net‐gunning (4 states), drop nets (4 states), Clover traps (3 states), and ground darting (4 states) (Clover 1956), with some states using multiple methods (Table 2). Similarly, we conducted all live‐capture and handling sessions during time periods that aligned with each state's policies.

Diagnostic samples included oral swab, nasal swab, and jugular/digital blood from each animal (Bevins et al. 2023). All swab samples were to be tested for the presence of SARS‐CoV‐2 by PCR at USDA National Wildlife Research Center's Wildlife Disease Diagnostics Laboratory (WDDL). Because SARS‐CoV‐2 serological assays are still being optimized for cervids, we quantified SARS‐CoV‐2 antibody titers by serial dilution using two separate methods: surrogate virus neutralization tests (sVNTs) conducted at WDDL, and conventional virus neutralization tests (cVNTs) conducted at Cornell University's Animal Health Diagnostic Center. The sVNT test was run by applying sVNT kits from both omicron and preomicron variant targets (Genscript cPassTM using the original HRP‐RBD and the HRP‐RBD Omicron variant B.1.1.529 applied separately following manufacturer's instructions). The cVNT was also run with targets to omicron and preomicron variants (Bewley et al. 2021). Collecting quantitative antibody titers is important for this project because it allows estimating time of infection for individual samples (e.g., as in (Pepin et al. 2017; Wilber et al. 2020) using captive animal infection kinetic data similar to (Hamer et al. 2022) and currently being collected for SARS‐CoV‐2 in white‐tailed deer in experiments at USDA's National Animal Health Center), which increases sample size for estimating population‐level epidemiological parameters that depend on current infection data as in (Hewitt, Wilson‐Henjum, Collins, Linder, et al. 2024; Hewitt, Wilson‐Henjum, Collins, Ringenberg, et al. 2024). Although the time of infection can theoretically be estimated from a single surveillance serum sample, uncertainty in the estimates can be reduced by collecting multiple samples from the same individuals over time.

## 3 Results

### 3.1 Network, Sites, and Data Collection Progress

The research network consists of over 50 scientists, ranging from technicians to professors and senior federal research scientists. Currently, the surveillance system consists of 22 sites in 9 states, including rural, suburban, and urban contexts across different ecoregions (Table 2). Specific states and target species include: Colorado (MuD & WTD), Illinois (WTD), Maryland/Washington D.C. (“MD/DC”; WTD), Minnesota (WTD), Nebraska (MuD), New York (WTD), Pennsylvania (WTD), Tennessee (WTD), and Utah (MuD) (Table 2). Eleven new sites were added in FY24 and one was discontinued for new GPS collar deployment. All sites continue to be sampled for SARS‐CoV‐2 exposure.

As of May 2024, we deployed 1304 GPS collars across 11 sites in fiscal year (FY) 2023 (October 2022 to September 2023) and 22 sites in FY24 (October 2023 to September 2024; Table 2), producing 213,156 days of GPS collar data thus far. Samples from collared animals were collected from 1068 adult females, 220 adult males, 154 juvenile females, and 100 juvenile males (Table S1). There have been 338 recaptures (Table S1). One thousand seven hundred seventy‐one samples have been tested for SARS‐CoV‐2 by PCR (results up to 1 May 2024), and 1753 samples have been tested for SARS‐CoV‐2 by sVNT. 288 of the 1753 samples (16.4%) were from hunter harvest or deer management (repeated cross‐sectional sampling that occurred only in MN, TN, and MD/DC) within the sites where GPS‐collared individuals occur, while the rest of the samples were from cohort sampling (Table 2).

### 3.2 Deviations From the Planned Design

Variation in ecological and regulation contexts resulted in some unavoidable deviations from our intended surveillance design at all sites (Box 1). For example, sites that primarily used helicopter capture (3 states, *n* = 10 sites) completed all captures and recaptures in distinct, short time windows (Table 2). This led to a sampling regime that included a few brief periods of intensive sampling with limited sampling in between. Sites that used ground darting, drop netting, and Clover trapping techniques (*n* = 12 sites) had longer sampling periods with less intensive sampling at each period. Capture timing also varied across sites according to habitat, land management type (public versus private), and state DNR policies.

BOX 1 **Examples of capture challenges encountered while implementing the landscape‐scale targeted surveillance system.**

Interannual variation in capture success

- Changes in capture personnel led to changes in capture success (IL)
- Undesirable environmental conditions (too little snow) led to cancelation of a capture event (MN)
- Changes in capture method across years led to different capture timing (TN)

Seasonal variation in capture success in some sites

- Summer recaptures were not feasible due to dense forage and cover, limiting sampling opportunities to winter or mortality events (PA, TN, IL)

Land access

- Study animals on private land could not be recaptured (NY and CO)

Human dimensions

- Appearance of deer with GPS collars resulted in public criticism requiring collars to be removed after the second sampling event (PA)

Other challenges

- Some sites had no evidence of exposure to SARS‐CoV‐2 (UT)
- Unreliable sample storage and shipping conditions from rural sites (UT)
- Partner constraints on aspects of data collection (GPS fix rate: UT; timing of sampling: TN).

Recapture success also varied across sites. Recapture was more difficult on sites with adjacent private lands, where captured individuals could not be accessed without landowner permission (Box 1). Summer recaptures were not possible at some sites due to abundant forage and cover or state DNR policies due to lactation. These challenges, and challenges with insufficient collar expansion over time on males, prevented standardization of capture and recapture timing across sites and introduced some site‐level variation in the sex ratios and age classes sampled that deviated from our planned design of equal sampling across sex and age classes (Table S1). Post‐mortem follow‐up provided an opportunity for an additional sampling occasion at many sites, leading us to establish methods for post‐mortem sampling analogous to the original antemortem sampling design.

Our initial sampling plan was designed to favor sites where all collars could be placed in a single patch. However, one of the states (MD/DC) that joined our network after establishment was working in an urban context that was fragmented with high deer densities, with ample opportunity to collect additional disease samples through repeated cross‐sectional surveillance. This elevated the need for a tool that could guide how to prioritize sample sizes within our diagnostic budget in a way that information would be optimized for our long‐term objectives. We addressed this challenge by developing a sample size estimator tool (available for use here: Sampling guidance tool; Data S1) based on sampling theory to guide decisions about how to allocate samples and collars in highly urban, fragmented landscapes (e.g., MD/DC sites).

### 3.3 Density Estimation

Our design did not specify particular methods for density estimation, although all sites were asked to provide some estimate of local density. Therefore, some states relied on existing local density estimation protocols (*n* = 3/9), some states established new procedures (*n* = 5/9), and some (*n* = 1/9) did not implement a density estimation method. Methods included annual aerial surveys (*n* = 1 state), universal or environmentally‐structured camera trapping or camera arrays (*n* = 5 states); forward‐looking infrared camera (FLIR) used while conducting roadside distance surveys (*n* = 1 state); or driven transect counts (*n* = 1 state). We are currently working on methods to integrate various density estimation methods across sites.

### 3.4 Testing for Additional Pathogens

Several sites established outside collaborations to screen serum or other sample types for other pathogens (non‐SARS‐CoV‐2) or specific antibodies (Table S1). Additional targets include: Chronic wasting disease prions (7/9 states), blue‐tongue virus (4/9 states), influenza A virus (7/9 states), epizootic hemorrhagic disease virus (3/9 states), Powassan virus (1/9 states), and metagenomic analysis with a viral panel (9/9 states). While the research network retains a voluntary list of diagnostics at each site to facilitate collaborative research on other pathogens, specific diagnostic assays and laboratories for additional pathogen testing are currently left to the particular sites involved.

### 3.5 GPS Data Collection and Management

We provided limited guidance about GPS collar specifications, beyond that we wanted a 30‐min fix rate and a battery life of 1–2 years. As a consequence, sites used a variety of different manufacturers, which has not posed major problems to date. One collar attribute that we should have specified is whether GPS data were uploaded continuously to a satellite or stored locally on the collar (i.e., stored “on‐board”). On‐board storage requires the biologist to get the collar back in hand to download the data. As a consequence, data from sites with store‐on‐board collars will not become fully available until the end of our current funding cycle, posing challenges for intermediate movement analyses that could have informed near‐term design decisions.

All sites format their GPS data for Movebank.org. Many sites use Movebank for GPS data storage, but not all sites had permission to use it; therefore, our workflows do not rely on Movebank per se. Instead, we constructed standardized workflows for GPS data preparation and cleaning (i.e., we tested that collar start and end dates matched capture and mortality, failure, or drop‐off dates for all collars; we checked for consistent projection information across all collars, we applied a standardized protocol for removing points with unreasonably “fast” round‐trips that indicate GPS fix errors, etc.). An across‐site working group meets periodically to discuss issues with GPS data preparation. Each site places cleaned GPS tracks onto github once every 6 months, and those cleaned tracks are shared with cross‐site analysts following registration of their proposed project and approvals from each site's PI.

In general, we have found that GPS analysis workflows must be adapted to specific sites due to cross‐site differences in deer movement behaviors, relevant barrier and resource types, and other drivers of deer movement ecology. Therefore, we are working to construct adaptable workflows that are structured enough to be efficiently reproducible but adaptable enough to capture relevant site‐specific differences. Thus far, we have constructed adaptable workflows for data cleaning, path segmentation to identify migratory or dispersal behaviors, covariate extraction via google earth engine, home range estimation via continuous time movement models, and habitat suitability analysis. Ultimately, our goal is to estimate and predict home range sizes, habitat suitability, landscape connectivity, and locations of intraspecific contact across all of our sites.

## 4 Discussion

### 4.1 Guidance for Deploying a Landscape‐Scale Targeted Surveillance Using a Research Network

Research network construction was expedited through existing collaborations that brought unique and necessary resources to the network at each stage of the surveillance system's deployment (Table 3). The federal agency employees provided leadership and coordination, centralized decision‐making about design and logistics, and standardized protocols and diagnostic testing across sites. University collaborators provided preexisting infrastructure through ongoing studies and well‐established relationships with local authorities to address permitting, land access, and local animal capture regulations. Rapid deployment required that university‐based partners already had strong working relationships with their state agencies and immediate availability (i.e., capacity to immediately shift a substantial amount of research time into logistical planning and deployment of this additional work). Local wildlife managers, land managers, and private landowners enhanced sampling by providing animal expertise and logistics management and engaging with local communities about sampling techniques (e.g., helicopter captures).

**TABLE 3:** Considerations and guidance for rapidly deploying landscape‐scale targeted surveillance in populations that are believed to be high‐risk for disease transmission and/or persistence.

Step	Research/site‐specific actions	Research comments	Administrative/centralized actions	Administrative comments
1. Build and resource the team	Identify and invite research collaborators. Establish contracts with partners. Initiate regular scientific meetings.	Institutions vary in infrastructure, costs, funding efficiencies, and expectations about researcher time. Consider using preexisting funding, activities, and expert networks where possible. Include disease, host, analytical, and field expertise.	Designate an administrative coordinator. Centralize sharing, permitting, and process documents. Identify stakeholder‐specific data privacy issues. Build data governance and sharing agreements.	Be prepared for system‐wide biosecurity training and permitting requirements. Be realistic about administrative burdens. Hire dedicated personnel who can handle both administrative and scientific tasks rapidly. Share information about contractors and contract language.
2a. Build the spatiotemporal sampling design	Determine sample size and repetition schedule. Specify desired age/sex structure and develop guidelines when the targeted structure is unattainable. Identify desired metadata to collect.	Site selection should account for local personnel skills, logistical challenges, and a desirable range of ecological contexts. Expect definitions of “site” to vary by institution. Aim to balance site‐specific and project‐wide objectives. Anticipate site‐specific wildlife capture regulations or constraints. Build template IACUC protocols to share across sites.	Communicate across sites re: capture timing and equipment. Initiate institution‐specific IACUC reviews. Build partnerships with agencies or individuals who hold jurisdiction over study site lands and animals.	Expect supply chain constraints re: animal tracking devices. Facilitate communication with capture contractors when multiple sites are using the same contractor, and consider aligning schedules. Make system‐wide decisions about whether sites should procure collars that store data on‐board or upload continuously.
2b. Build the disease sampling design	Identify appropriate specimens for diagnostic testing.	Diagnostic testing capabilities may change over the course of the study. The timeline from sample submission to reported results may exceed time between sampling events, especially if diagnostic tests are under development or supply chains are strained. Build a standardized necropsy plan prior to captures.	Centralize deployment of sampling supplies, sampling kits, and sample management. Build and share standard operating procedures for sample collection, storage, and shipping. Establish shipping accounts with major shippers. Identify and order sparse sampling supplies.	Expect supply chain constraints re: disease testing supplies, particularly during pandemics. Consider stockpiling basic supplies with partners ahead of time. Consider using a centralized sample labeling scheme that includes barcodes.
3. Collect data	Hire dedicated field staff. Capture and sample animals. Appropriately store and ship specimens. Manage and share metadata. Share tips, tricks, and emergent problems with other sites.	Sites will vary in how well they hit design targets, and in their ability to store and ship samples under desired conditions. Hold regular researcher meetings for boots‐on‐the‐ground information sharing. Identify local labs for support.	Build consistent communication protocols around sample shipment. Develop a centralized data management system (e.g., Open Science Forum). Organize stored samples and coordinate diagnostic testing. Have sample management plans in place at diagnostic facilities.	Know the required sample volume for all assays and consider gathering multiple aliquots per animal. Leverage existing data collation platforms (e.g., Movebank or other cloud storage platforms).
4. Analyze data	Identify and discuss analytical products. Propose specific analyses on local or cross‐site publicly. Provide analysts with perspective across sites. Build topical working groups across collaborating sites to maintain consistency.	Analyses will vary in computational time, and may not always be replicable across all sites. Identify time consuming but rote tasks, and centrally hire individuals to conduct those tasks across all sites. Build junior researcher teams to facilitate cross‐site communication. Maintain regular communication about research progress among PIs.	Implement a structure for preregistering analyses and formalizing analysis‐specific data sharing. Share data according to the permissions scheme with appropriate analysts. Collate analytical output into a project‐wide portfolio or final report.	Develop, modularize, and automate data flows for updating laboratory results and animal tracking data, and linking those data to metadata.
5. Communicate results	Develop site‐specific communication plans to build local awareness	Different sites may prioritize communication to different audiences, and the relative importance of institutional, local, and national stake‐holders will vary across sites. Provide consistent, timely reporting. Allow flexibility for additional objectives.	Clarify unified messaging for national‐scale stakeholders. Develop within‐network communication plan to communicate progress and release results. Develop plans to engage additional partners: e.g., short proposal process for joining the network (i.e., becoming a new site). Leverage network for additional sample collection or cross‐site analyses	Establish team‐wide press and communication strategies. It might take several weeks to clear public communications with all stakeholders.
6. Build system sustainability	Consider funding opportunities that could maintain the collection of core data streams. Identify site capacities to maintain some level of sample collection, banking, and storage after the project ends. Consider banking samples throughout the project to allow for a broader scope of future questions.	Researcher schedules and interests will lead to some changes in the team through time.	Identify the minimal sampling requirements necessary to maintain core aspects of the system. Consider economic bottlenecks and plan appropriately.	Different sites will vary in their ability to sustain projects beyond the initial timeline. Build relationships among collaborating partners with in‐person meetings where possible. Acquire infrastructure to allow for low‐cost, longer‐term data collection to cover gaps in funding.

Attracting and maintaining surveillance network partners was made easier by allowing individual partners to pursue additional research interests outside the immediate scope of the landscape‐scale targeted surveillance objectives. These additional research directions synergized with disease surveillance activities by providing additional data collection that increased sample size, improved knowledge of host demographics, and enabled new avenues for epidemiological research (*sensu* (Cardoso et al. 2022)). Multiple objectives that appeal to an array of stakeholders is a lynchpin of system sustainability, since surveillance systems that serve multiple purposes can appeal to a wider range of potential funders and thus be more robust to changing fiscal allocations. In our case, data related to deer ecology, density, health, behavior, population dynamics, and pathogen evolution brought additional resources at some sites due to alignment with other research questions and programs.

We aimed to standardize within‐site design elements across multiple sites. Important considerations included determining sample size and schedule, prioritizing demographic targets (e.g., optimal age/sex structure), establishing diagnostic protocols, and identifying desirable metadata and landscape‐level spatial covariates. While these decisions are straightforward in highly controlled livestock systems, some were less obvious or less attainable when we applied them to wildlife. Involving partners with on‐the‐ground experience in different environments proved critical for identifying appropriately flexible standards, while maintaining critical design elements across sites so that data could be compared within a common analytical framework. Adapting the overall design to accommodate the realities of each site required regular structured discussions and decision‐making during the design phase.

Centralized deployment of resources and data management structures expedited the roll‐out of sampling efforts, while also balancing logistical burdens so that site‐specific personnel could focus on capture and sampling. Centralized organization and deployment of supplies, protocols, instructions, and datasheets also enabled rapid adaptation to unexpected challenges and easy addition of new sample types during ongoing field operations. Centralized supply preparation and data management also prevented ad hoc, on‐the‐fly changes that could negatively impact overall productivity or data standardization.

The heterogeneous capture schedules that arose across sites may ultimately improve our ability to describe SARS‐CoV‐2 disease dynamics. When samples are collected at low rates over long periods, the sample size for any given time period is small, but the data can capture rapid swings in pathogen prevalence if those changes are large. In contrast, larger sample sizes collected at fewer occasions can identify smaller changes in prevalence, but at a slower timescale. Both sets of information have unique value, and we intend to meld them by accounting for sampling design differences in mechanistic disease‐dynamic models that use data from all sites.

### 4.2 Data Analytics

As of now, we have shifted focus to coordination and delivery of data analytics. Our sampling design and emergent data provide a groundbreaking dataset for advancing methods of inference in wildlife disease ecology (Figure 2). Recent theoretical developments that combine cutting‐edge inferential tools in movement ecology (e.g., continuous‐time movement models, resource‐driven utilization distributions, conditional distribution of encounters; (Wilber et al. 2022; Hewitt, Wilson‐Henjum, Collins, Ringenberg, et al. 2024; Vargas Soto et al. 2024; Viana et al. 2014; Calabrese et al. 2016; Noonan et al. 2021; Potts and Börger 2023)) and epidemiological theory provide the means to link observed movement data with transmission risk on real‐world landscapes (Manlove et al. 2022; Merkle et al. 2018; Yang et al. 2023; Vargas Soto et al. 2024). Combining pathogen genomic data with host ecology and epidemiological data streams allows for tracking adaptive changes in pathogens and linking them to ecological drivers (McBride et al. 2023). Recently, we began conducting metagenomic analyses (Wittekindt et al. 2010; Simner et al. 2018; Mwakibete et al. 2024) with a virus panel on samples from all sites to evaluate whether other pathogens are present at higher frequencies than SARS‐CoV‐2 and to better understand coinfections in deer. The metagenomic analyses will complement targeted genomic sequencing by offering unbiased universal pathogen detection and detecting patterns of coinfection or viral sharing. Pairing targeted genomic sequencing and metagenomic data with our GPS movement and contact analyses and other diagnostic data will help to understand which patterns of contact lead to pathogen transmission.

### 4.3 Building Surveillance System Sustainability

Initially, the targeted surveillance system received 3 years of funding in two separate blocks. Almost immediately, sites began pursuing complementary funding or partnerships that will allow some aspects of the surveillance system to continue running past the end of that period. As the original funded period draws to a close, we are working together to (1) establish a minimum sample, collar, and metadata list for our sites and others to continue working; (2) understand each site's capacity to bank samples into the future and plan accordingly; and (3) collaboratively procure funding to continue the system. The end of this funding block will bring an end to dedicated time from the federal employees; replacing that effort or finding a way for the sites to continue in some capacity without dedicated coordination is perhaps the largest impediment to system sustainability.

### 4.4 Broader Impacts

Long‐term monitoring systems are essential for understanding ecological systems (Cardoso et al. 2022). The principles proposed here are apparent in other national‐scale surveillance systems across disciplines, such as the National Ecological Observation Network (NEON) and the U.S. and International Long Term Ecological Research (LTER and ILTER) networks (Van Vanderbilt and Gaiser 2017; Hobbie et al. 2003), each of which coordinates long‐term environmental data across diverse ecosystems to understand the impacts of environmental change on ecosystem function (Van Vanderbilt and Gaiser 2017; Hobbie et al. 2003). Long‐term monitoring is not solely the purview of the federal government, however: for example, the Nutrient Network (“NutNet”) is an academic‐driven research network that consists of a fundamental experimental design surrounded by locally flexible elements to understand grassland community dynamics worldwide (Borer et al. 2017). Such landscape‐scale targeted surveillance that standardizes data collection across several scales of biological organization is needed to gain a mechanistic understanding that can support the prediction of new disease emergence events in wildlife species or at the wildlife‐human interface. Indeed, previous work from national‐scale opportunistic surveillance of SARS‐CoV‐2 in WTD has been limited to characterizing spillover patterns or identifying potential ecological risk factors (e.g., (Feng et al. 2023; Hewitt, Wilson‐Henjum, Collins, Linder, et al. 2024)) instead of providing inference about which ecological conditions lead to different epidemiological outcomes in terms of outbreak severity or persistence. Our landscape‐scale targeted surveillance approach provides data collected on the scale of transmission between individuals, populations, and across ecological contexts, thus allowing for inferences about particular ecological contexts and host ecology on epidemiological outcomes.

The expense and complexity of monitoring wildlife diseases (Pruvot et al. 2023) in a way that allows for both inference and prediction (Plowright et al. 2019) have motivated several efforts to recommend and improve study designs (e.g., (Nusser et al. 2008; Walton et al. 2016; ENETWILD‐Consortium et al. 2023; Hoye et al. 2010)). Some have called for (adaptive) risk‐based surveillance (e.g., (Miller et al. 2022; Viljugrein et al. 2021)) or other computational approaches for optimizing surveillance designs (e.g., (Belsare et al. 2020; Cheng et al. 2020)) to increase the efficiency of sampling that will be informative for predicting disease in wildlife. However, these recommendations may call for surveillance designs that are infeasible or ultimately confound implementation on the group. We demonstrate how to tackle some challenges associated with implementing large‐scale surveillance designs in wildlife that move beyond opportunistic sampling by targeting epidemiological processes across the scales of individuals, populations, and landscapes (Figure 1, Table 3).

Previous work suggested that coordinated wildlife disease surveillance networks of researchers and other partners could overcome the logistical and financial challenges of robust wildlife disease surveillance (Watsa 2020; Pruvot et al. 2023). We demonstrate how the operational public service and research sectors can be integrated to build comprehensive workflows for surveillance in high‐risk wildlife disease reservoirs—an important gap for addressing complex disease emergence challenges (Pepin et al. 2024). Our approach combines the strengths of both academic and public service partners—the operational and coordination capabilities of public service partners, and the rigorous scientific approach and contextual expertise of research institutions. Once established, disease surveillance research networks can be leveraged to provide information about other pathogens or parasites, provide data for analytic methods development, or be readily adapted for deployment in sympatric host species.

Wild deer are a highly abundant and valued species in the U.S. and can introduce and transmit important diseases of interest to wildlife management, wildlife health, and conservation, agriculture, and public recreation. While our network was constructed for understanding the emergence and persistence of SARS‐CoV‐2 in wild deer, it is also being leveraged to improve risk assessment for endemic cervid diseases such as chronic wasting disease, epizootic hemorrhagic disease, and bluetongue, and to evaluate whether deer may play a role in the rapidly changing ecology and emergence of current influenza A variants across the U.S. Similarly, some partners in our network are leveraging the system to better understand emerging wildlife health issues such as the impacts of perfluoroalkyl and poly‐fluoroalkyl substances (PFAS). Our approach of collecting detailed host ecology data alongside pathogen data provides information for understanding habitat‐specific disease occurrence and transmission processes. This type of mechanistic understanding can be used to inform effective control points for disease management.

Our work describes a large simultaneous deployment of disease testing and GPS collaring in many free‐ranging terrestrial populations across very diverse landscapes. This type of dataset is rare, proving unique information for understanding drivers of animal movement that determine disease introduction, establishment, and/or persistence. Here, we focus on how to overcome challenges with implementing a study where the goal is to coordinate the same design in wildlife populations in very different ecological contexts. The data being generated by this network can be leveraged for advancing methods development and applications in wildlife behavior and animal movement (e.g., (Manlove et al. 2022; Dougherty et al. 2018; Yang et al. 2021; Noonan et al. 2021; Getz and Saltz 2008; McGarigal et al. 2016)). Understanding how wildlife use and move through different habitats and how habitat feeds back to inform individual status and local densities are fundamental for anticipating and managing human‐wildlife conflict or emerging disease (including foreign animal diseases or spillovers in wildlife host species) and for delineating appropriate ecosystem reserves for conservation. Our surveillance system provides a rich source of data to inform those efforts through its integrative approach to surveilling individual, population, and landscape‐scale processes concurrently.

## Author Contributions

**Kim M. Pepin:** conceptualization (lead), formal analysis (equal), funding acquisition (lead), investigation (equal), methodology (equal), project administration (lead), resources (equal), supervision (lead), visualization (equal), writing – original draft (lead), writing – review and editing (equal). **Matthew A. Combs:** visualization (equal), writing – original draft (equal), writing – review and editing (equal). **Guillaume Bastille‐Rousseau:** conceptualization (equal), data curation (equal), investigation (equal), methodology (equal), resources (equal), supervision (equal), writing – review and editing (equal). **Meggan E. Craft:** conceptualization (equal), data curation (equal), investigation (equal), methodology (equal), project administration (equal), resources (equal), supervision (equal), writing – review and editing (equal). **Paul Cross:** conceptualization (equal), methodology (equal), writing – review and editing (equal). **Maria A. Diuk‐Wasser:** investigation (equal), project administration (equal), resources (equal), supervision (equal), writing – review and editing (equal). **Roderick B. Gagne:** investigation (equal), methodology (equal), resources (equal), supervision (equal), writing – review and editing (equal). **Travis Gallo:** funding acquisition (equal), investigation (equal), methodology (equal), resources (equal), writing – review and editing (equal). **Tyler Garwood:** data curation (equal), investigation (equal), writing – original draft (equal), writing – review and editing (equal). **Jonathon D. Heale:** funding acquisition (supporting), project administration (supporting), writing – review and editing (supporting). **Joshua Hewitt:** formal analysis (equal), software (equal), writing – review and editing (equal). **Jennifer Høy‐Petersen:** investigation (supporting), visualization (supporting), writing – original draft (supporting), writing – review and editing (supporting). **Jennifer Malmberg:** funding acquisition (equal), investigation (equal), resources (equal), writing – review and editing (supporting). **Jennifer Mullinax:** funding acquisition (equal), investigation (equal), methodology (equal), resources (equal), writing – review and editing (supporting). **Laura Plimpton:** investigation (supporting), methodology (supporting), writing – review and editing (supporting). **Lauren Smith:** project administration (supporting), visualization (supporting), writing – review and editing (supporting). **Meredith C. VanAcker:** conceptualization (equal), investigation (equal), methodology (equal), project administration (equal), resources (equal), writing – review and editing (equal). **Jeffrey C. Chandler:** formal analysis (lead), resources (equal), supervision (equal), writing – review and editing (supporting). **W. David Walter:** conceptualization (equal), investigation (equal), methodology (equal), project administration (equal), resources (equal), supervision (equal), writing – review and editing (equal). **Grete Wilson‐Henjum:** data curation (lead), project administration (supporting), writing – review and editing (supporting). **George Wittemyer:** conceptualization (equal), investigation (equal), methodology (equal), project administration (equal), resources (equal), supervision (equal), writing – review and editing (equal). **Kezia Manlove:** conceptualization (equal), investigation (equal), methodology (equal), project administration (equal), resources (equal), supervision (equal), writing – original draft (equal), writing – review and editing (equal).

## Disclosure

Statement of inclusion: Our study brings together authors from across the U.S. where the study was conducted. Authors were engaged at the project initiation phase or as they demonstrated interest in participating. Weekly or bi‐weekly virtual meetings were held for project coordination.

## Conflicts of Interest

The authors declare no conflicts of interest.

## Supporting information


Data S1.

Table S1.

## Data Availability

All relevant data and metadata for replicating our study design are contained in the body of the manuscript. This study did not involve computer code. This manuscript describes an after‐action review of a study design for deploying a national‐scale surveillance system through a public‐private partnership.
