# Supplementary material for: Expanding National‐Scale Wildlife Disease Surveillance Systems With Research Networks
Source: Ecol Evol. 2025 Jun 11;15(6):e71492. doi: 10.1002/ece3.71492 (PMC12158667; doi:10.1002/ece3.71492)
Supplement: Supplementary file 1 — Data S1. [file ECE3-15-e71492-s001.pdf]

# Supplement to "Expanding national-scale wildlife disease intelligence systems with research networks": A model to evaluate simple wildlife disease surveillance sampling designs

Kim M. Pepin[1*], Matthew A. Combs[1], Guillaume Bastille-Rousseau[2], Meggan E. Craft[3], Paul Cross[4], Maria Diuk-Wasser[5], Roderick B. Gagne[9], Travis Gallo[10], Tyler Garwood[3], Jonathon D. Heale[7], Joshua Hewitt[1,8], Jennifer Høy-Petersen[9], Jennifer Malmberg[1], Jennifer Mullinax[10], Laura Plimpton[5], Lauren Smith[1,8], Meredith C. VanAcker[5,6], Kurt VerCauteren[1], Jefferey C. Chandler[1], W. David Walter[11], Grete Wilson-Henjum[1,8], George Wittemyer[12], Kezia Manlove[8]

[1]National Wildlife Research Center, Wildlife Services, Animal and Plant Health Inspection Service, United States Department of Agriculture, Fort Collins, CO, 80523, USA
[2]Cooperative Wildlife Research Laboratory, Southern Illinois University, Carbondale, IL, 62901, USA
[3]Department of Ecology, Evolution, and Behavior, University of Minnesota, St. Paul MN, USA
[4]Cross affiliation
[5]Department of Ecology, Evolution, and Environmental Biology, Columbia University, New York City, NY, USA
[6]Global Health Program, Smithsonian National Zoo and Conservation Biology Institute, Washington, DC, USA
[7]Heale affiliation
[8]Department of Wildland Resources and Ecology Center, Utah State University, Logan, UT, 84341, USA
[9]Department of Pathobiology, Wildlife Futures Program, University of Pennsylvania School of Veterinary Medicine, Kennett Square, PA 19348, USA
[10]Department of Environmental Science and Technology, University of Maryland, College Park, MD, 20742, USA
[11]U.S. Geological Survey, Pennsylvania Cooperative Fish and Wildlife Research Unit, The Pennsylvania State University, University Park, Pennsylvania, 16802, USA
[12]Department of Fish, Wildlife and Conservation Biology, Colorado State University, Fort Collins, CO, 80523, USA
[*]Corresponding author: kim.m.pepin@usda.gov

## 1 Motivation

Finite sampling methods can estimate the prevalence of a characteristic in a finite population while accounting for sample size, such as the proportion of a population that has a disease. Finite sampling methods compare the number of sampled individuals to the size of the population. The prevalence estimate's uncertainty decreases as the sample gets closer to a census, in which the status of all individuals is known (i.e., whether an individual has a disease or not). However, the population size is often unknown for wildlife populations. Additional complications arise for wildlife disease surveys, in which the status of an individual animal may not be perfectly known due to imperfect diagnostic tests. We describe a basic model that can be used to design studies and estimate prevalence for wildlife disease when 1) sampling a single, finite, density-based population with fixed prevalence of a characteristic, 2) the population is only sampled once (i.e., precluding capture-recapture sampling designs), and 3) the observed status of sampled animals is subject to false positive and false negative identification errors.

## 2 Model specification

We specify a simple Bayesian model for a characteristic within a finite population at a single point in time or some other equilibrium point. We assume an unknown population density $\lambda > 0$ modeled via

$$\lambda \sim \Gamma\left(a^{(\lambda)}, b^{(\lambda)}\right),$$

in which $\Gamma(a^{(\lambda)}, b^{(\lambda)})$ represents a Gamma distribution parameterized to have prior expected value $\mathrm{E}[\lambda] = a^{(\lambda)}/b^{(\lambda)}$. The density $\lambda$ specifies a Poisson process that models the unknown population size $N \in \mathbb{N}$ via

$$N|\lambda \sim \text{Poisson}\left(\lambda A\right),$$

in which $A$ is the known area of the site in which the population resides. We assume the status of each animal in the population is conditionally independent from other animals. The fixed prevalence $p \in [0, 1]$ for the characteristic is modeled via

$$p \sim \text{Beta}\left(a^{(p)}, b^{(p)}\right),$$

which defines the risk that each animal has the characteristic. The random variable $X_i$ for $i = 1, \ldots, N$ indicates whether the $i$th animal has the characteristic. The variable $X_i$ is modeled via

$$X_i|p \sim \text{Bernoulli}(p).$$

We assume each animal is equally likely to be sampled from the population and $N_{\mathcal{S}} < N$ animals are sampled. Sensitivity $\varphi \in [0, 1]$ models the probability that an animal with the characteristic is correctly identified (i.e., the true positive rate). Similarly, specificity $\phi \in [0, 1]$ models the probability that an animal without the characteristic is correctly identified (i.e., the true negative rate). Sensitivity and specificity are modeled via

$$\varphi \sim \text{Beta}\left(a^{(\varphi)}, b^{(\varphi)}\right),$$
$$\phi \sim \text{Beta}\left(a^{(\phi)}, b^{(\phi)}\right).$$

The sampling and test error assumptions imply the observed test result $Y_i$ for the $i$th animal is distributed via

$$Y_i|X_i, \varphi, \phi \sim \text{Bernoulli}\left(\varphi X_i + (1-\phi)(1-X_i)\right).$$

Marginally, the total number of positive test results $Y_{\mathcal{S}} = \sum_{i=1}^{N_{\mathcal{S}}} Y_i$ in the sample is distributed via

$$Y_{\mathcal{S}}|N_{\mathcal{S}}, \varphi, \phi, p \sim \text{Binomial}\left(N_{\mathcal{S}}, \varphi p + (1-\phi)(1-p)\right),$$

which serves as a sufficient statistic for the information contained in the sample about population-level parameters.

The likelihood for the model is specified via

$$\ell(\varphi, \phi, p, N, \lambda; Y_{\mathcal{S}}, N_{\mathcal{S}}) = [Y_{\mathcal{S}}|N_{\mathcal{S}}, \varphi, \phi, p][\varphi][\phi][p][N_{\mathcal{S}}|N][N|\lambda][\lambda],$$

in which $[\cdot]$ represents probability density and mass functions, as appropriate. We assume the distribution $[N_{\mathcal{S}}|N]$ is improper, simply representing a constraint that $N_{\mathcal{S}} < N$. Naturally, the distribution $[N_{\mathcal{S}}|N]$ may be more complex, potentially representing sampling effort.

## 3 Posterior sampling and computation

We use the posterior distribution $[\varphi, \phi, p, N, \lambda|Y_{\mathcal{S}}, N_{\mathcal{S}}]$ to draw inference on the true, finite-population prevalence of the characteristic in the population and other parameters. We use Markov-Chain Monte Carlo

(MCMC) methods to generate a sample from $[\varphi, \phi, p, N, \lambda | Y_{\mathcal{S}}, N_{\mathcal{S}}]$. Standard, Metropolis-Hastings random walk proposal distributions and slice samplers are used when conjugate updates are not possible.

The finite-population prevalence $p_f$ is the number of animals with the characteristic divided by the population size, specified via

$$p_f = T/N,$$

in which $T = \sum_{i=1}^{N} X_i$ represents the number of animals with the characteristic in the population. We approximate the posterior distribution for the finite-population prevalence $[p_f | Y_{\mathcal{S}}, N_{\mathcal{S}}]$ via composition sampling the joint posterior distribution $[T, N | Y_{\mathcal{S}}, N_{\mathcal{S}}]$. Composition sampling computes $p_{f_m} = T_m/N_m$ for $m = 1, \ldots, M$ using samples $(T_m, N_m)$ drawn from $[T, N | Y_{\mathcal{S}}, N_{\mathcal{S}}]$. Sample averages of the composition samples $p_{f_1}, \ldots, p_{f_M}$ form Monte Carlo approximations of expectations for the target posterior distribution $[p_f | Y_{\mathcal{S}}, N_{\mathcal{S}}]$.

The posterior distribution $[T, N | Y_{\mathcal{S}}, N_{\mathcal{S}}]$ can be decomposed via

$$\begin{aligned}[T, N | Y_{\mathcal{S}}, N_{\mathcal{S}}] &= \int [T, N, X_{\mathcal{U}}, X_{\mathcal{TP}}, X_{\mathcal{FN}}, p, \varphi, \phi | Y_{\mathcal{S}}, N_{\mathcal{S}}] dX_{\mathcal{U}} dX_{\mathcal{TP}} dX_{\mathcal{FN}} dp d\varphi d\phi \\ &= \int [T | X_{\mathcal{U}}, X_{\mathcal{TP}}, X_{\mathcal{FN}}][X_{\mathcal{U}}, X_{\mathcal{TP}}, X_{\mathcal{FN}} | p, \varphi, \phi, N, Y_{\mathcal{S}}, N_{\mathcal{S}}][N, p, \varphi, \phi | Y_{\mathcal{S}}, N_{\mathcal{S}}] dX_{\mathcal{U}} dX_{\mathcal{TP}} dX_{\mathcal{FN}} dp d\varphi d\phi,\end{aligned} \tag{1}$$

in which $X_{\mathcal{U}}$ represents the number of animals with the characteristic in the unsampled population, $X_{\mathcal{TP}}$ represents the number of animals with true-positive test outcomes in the sample, and $X_{\mathcal{FN}}$ represents the number of animals with false-negative test outcomes in the sample. The decomposition (1) suggests a composition sampling scheme. First, posterior samples from $[\varphi, \phi, p, N, \lambda | Y_{\mathcal{S}}, N_{\mathcal{S}}]$ can be used as composition samples for $[N, p, \varphi, \phi | Y_{\mathcal{S}}, N_{\mathcal{S}}]$. Next, $[T | X_{\mathcal{U}}, X_{\mathcal{TP}}, X_{\mathcal{FN}}]$ is a degenerate distribution since $T = X_{\mathcal{U}} + X_{\mathcal{TP}} + X_{\mathcal{FP}}$ by definition. Finally, $[X_{\mathcal{U}}, X_{\mathcal{TP}}, X_{\mathcal{FN}} | p, \varphi, \phi, N, Y_{\mathcal{S}}, N_{\mathcal{S}}]$ can be sampled directly.

The distribution $[X_{\mathcal{U}}, X_{\mathcal{TP}}, X_{\mathcal{FN}} | p, \varphi, \phi, N, Y_{\mathcal{S}}, N_{\mathcal{S}}]$ decomposes as the product of three conditionally independent binomial random variables. The decomposition is specified via

$$[X_{\mathcal{U}}, X_{\mathcal{TP}}, X_{\mathcal{FN}} | p, \varphi, \phi, N, Y_{\mathcal{S}}, N_{\mathcal{S}}] = [X_{\mathcal{U}} | p, N, N_{\mathcal{S}}][X_{\mathcal{TP}} | p, \varphi, \phi, Y_{\mathcal{S}}][X_{\mathcal{FN}} | p, \varphi, \phi, Y_{\mathcal{S}}, N_{\mathcal{S}}],$$

which we derive from properties of the full posterior distribution at the animal level $[X_1, \ldots, X_N | p, \varphi, \phi, Y_1, \ldots, Y_{N_{\mathcal{S}}}, N]$ in the next paragraph. The binomial distributions are specified via

$$\begin{aligned} X_{\mathcal{U}} | p, N, N_{\mathcal{S}} &\sim \text{Binomial}\left(N - N_{\mathcal{S}}, p\right), \\ X_{\mathcal{TP}} | p, \varphi, \phi, Y_{\mathcal{S}} &\sim \text{Binomial}\left(Y_{\mathcal{S}}, \pi^+/(\pi^+ + \pi^-)\right), \\ X_{\mathcal{FN}} | p, \varphi, \phi, Y_{\mathcal{S}}, N_{\mathcal{S}} &\sim \text{Binomial}\left(N_{\mathcal{S}} - Y_{\mathcal{S}}, (p - \pi^+)/(1 - \pi^+ - \pi^-)\right), \end{aligned}$$

in which $\pi^+ = \varphi p$ and $\pi^- = (1 - \phi)(1 - p)$.

The conditional independence and binomial results follow from properties of $[X_1, \ldots, X_N | p, \varphi, \phi, Y_1, \ldots, Y_{N_{\mathcal{S}}}, N]$. The distribution decomposes via

$$\begin{aligned} [X_1, \ldots, X_N | p, \varphi, \phi, Y_1, \ldots, Y_{N_{\mathcal{S}}}, N] &\propto \prod_{i=1}^{N_{\mathcal{S}}} [Y_i | X_i, \varphi, \phi][X_i | p] \prod_{i=N_{\mathcal{S}}+1}^{N} [X_i | p] \\ &\propto \prod_{i=1}^{N_{\mathcal{S}}} [X_i | Y_i, \varphi, \phi, p] \prod_{i=N_{\mathcal{S}}+1}^{N} [X_i | p], \end{aligned}$$

which is a product of conditionally independent Bernoulli random variables. The conditionally independent random variables $X_i | Y_i, \varphi, \phi, p$ and $X_i | p$ can be aggregated into the binomial random variables $X_{\mathcal{TP}}$ and $X_{\mathcal{FN}}$ according to the observed values of $Y_i$ for the sampled animals, and into $X_{\mathcal{U}}$ for the unsampled animals, respectively. The success probability for $X_{\mathcal{U}}$ is based on the population's generative distribution $[X_i | p]$. The success probabilities for $X_{\mathcal{TP}}$ and $X_{\mathcal{FN}}$ can be evaluated via Bayes rule, which specifies

$$P(X_i = x | Y_i = y, p, \varphi, \phi) = \frac{P(Y_i = y | X_i = x, \varphi, \phi) P(X_i = x | p)}{\sum_{x' \in \{0,1\}} P(Y_i = y | X_i = x', \varphi, \phi) P(X_i = x' | p)},$$

simplifying to

$$P(X_i = 1|Y_i = 1, p, \varphi, \phi) = \frac{\varphi p}{\varphi p + (1-\phi)(1-p)} = \frac{\pi^+}{\pi^+ + \pi^-},$$
$$P(X_i = 1|Y_i = 0, p, \varphi, \phi) = \frac{(1-\varphi)p}{(1-\varphi)p + \phi(1-p)} = \frac{p - \pi^+}{1 - \pi^+ - \pi^-}.$$

# 4 Potential applications

The model and posterior approximations can evaluate both sampling designs and outcomes. The posterior distribution can directly analyze disease survey results. Sampling designs can also be analyzed before data collection. To do so, different values for $Y_{\mathcal{S}}$, $N_{\mathcal{S}}$, and other prior distribution parameters can be entered to explore the potential analytic findings that different study designs and outcomes would yield. In particular, parameters for prior distributions can be changed to represent what might happen if a low-prevalence population was sampled with a low-sensitivity assay.

The posterior distribution can address many applied questions. For example, the marginal posterior distribution $[N|Y_{\mathcal{S}}, N_{\mathcal{S}}]$ represents population size estimates based on the number of samples taken. Similarly, scaling $N_{\mathcal{S}}$ by posterior samples for $N$ will yield composition samples that estimate the fraction of the population that was sampled. Higher values of the fraction indicate higher chances that the population was close to being censused. The marginal posterior distribution $[p_f|Y_{\mathcal{S}}, N_{\mathcal{S}}]$ estimates likely values or upper bounds for finite-population prevalence after data collection. If $Y_{\mathcal{S}} = 0$, then the posterior probability $P(p_f = 0|Y_{\mathcal{S}}, N_{\mathcal{S}})$ is an estimate of the chance the population is free from disease.
